# Preventing inadvertent drain removal using a novel catheter securement device

**DOI:** 10.1038/s41598-023-37850-2

**Published:** 2023-09-26

**Authors:** Mario Russo, John Di Capua, April Anlage, Hersh Bendre, Jon Kusner, Graham Lieberman, Sean Jang, Zubin Irani, Ronald S. Arellano, Patrick D. Sutphin, Sara Smolinski-Zhao, Dania Daye, Sanjeeva P. Kalva, Marc D. Succi, Avik Som, Ashraf Thabet

**Affiliations:** 1 Harvard Medical School, 25 Shattuck Street, Boston, MA 02115 USA; 2 Massachusetts General Hospital, 55 Fruit Street, Boston, MA 02114 USA; 3 Massachusetts Institute of Technology, 77 Massachusetts Ave, Cambridge, MA 02139 USA; 4 Duke University, 2301 Erwin Rd, Durham, NC 27710 USA; 5 Device Division, Massachusetts General Hospital, Medically Engineered Solutions in Healthcare Incubator (MESH), Boston, MA USA; 6 Department of Radiology, Massachusetts General Hospital, Boston, MA USA

**Keywords:** Disease prevention, Translational research, Health care, Medical research

## Abstract

Percutaneous drains have provided a minimally invasive way to treat a wide range of disorders from abscess drainage to enteral feeding solutions to treating hydronephrosis. These drains suffer from a high rate of dislodgement of up to 30% resulting in emergency room visits, repeat hospitalizations, and catheter repositioning/replacement procedures, which incur significant morbidity and mortality. Using ex vivo and in vivo models, a force body diagram was utilized to determine the forces experienced by a drainage catheter during dislodgement events, and the individual components which contribute to drainage catheter securement were empirically collected. Prototypes of a skin level catheter securement and valved quick release system were then developed. The system was inspired by capstans used in boating for increasing friction of a line around a central spool and quick release mechanisms used in electronics such as the Apple MagSafe computer charger. The device was tested in a porcine suprapubic model, which demonstrated the effectiveness of the device to prevent drain dislodgement. The prototype demonstrated that the miniaturized versions of technologies used in boating and electronics industries were able to meet the needs of preventing dislodgement of patient drainage catheters.

## Introduction

Minimally invasive percutaneous drainage catheters have revolutionized the treatment of a wide variety of diseases, ranging from infectious abdominal complications, such as perforated appendicitis or diverticulitis, to decompression of obstructed urinary tracts from kidney stones to providing enteral access for patients with neurodegenerative disorders. The use of percutaneous drainage catheters has converted previously lethal illnesses treated with morbidly invasive procedures to chronic, palliative, or often curable disease states by providing a small outlet or inlet to previously inaccessible regions of the body. Unfortunately, a common complication plagued by all percutaneous drainage catheters is accidental catheter dislodgement (ACD)^1–3^.

**Figure 1:**
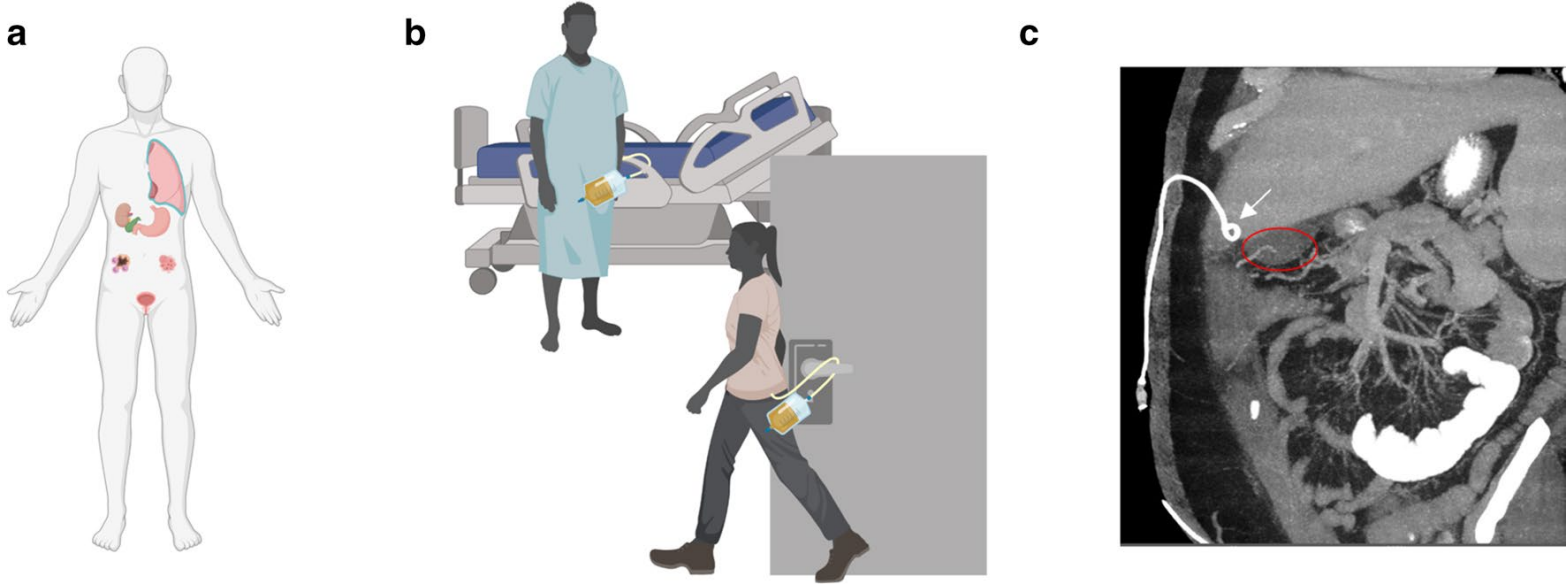
Clinical Scenario—(**A**) Illustration of the variety of locations drainage catheters are used, including pleura, intrahepatic, gastric, bladder, malignant and infectious foci; (**B**) demonstration of common dislodgement situations; (**C**) computed tomography (CT) imaging demonstrating a pigtail catheter (white arrow) after it has been unintentionally retracted from its intended drainage site, the gallbladder (red oval). Figure 1a-b produced using www.BioRender.com Individual License and Fig. 1c is exported from Visage 7.

Percutaneous drainage catheters may either be partially or completely dislodged from the patient. This typically takes place when a pull force is applied to the external component of the device. This may occur during sleep, normal daily activities, or self-removal in the case of patients with altered mental status (Fig. 1)^1,2,4,5^ Partial catheter dislodgement may result in decreased drainage catheter output while the dislodgement may not be as externally apparent to the patient or healthcare provider (Fig. 1C).

The risks associated with ACD depend on the type of catheter dislodged, but are typically related to tissue trauma during the dislodgement event, interruption in necessary device function, and risks related to reinsertion^3^ Each of these complications are exacerbated by disparities in healthcare access and patient distance from requisite specialty care. In addition to these physical harms, management of patients who experience ACD poses significant financial burdens. ACD is associated with costs related to emergency department utilization, radiologic imaging, specialty procedural care, inpatient admission, and prolonged length of hospital stay^6,7^

**Figure 2:**
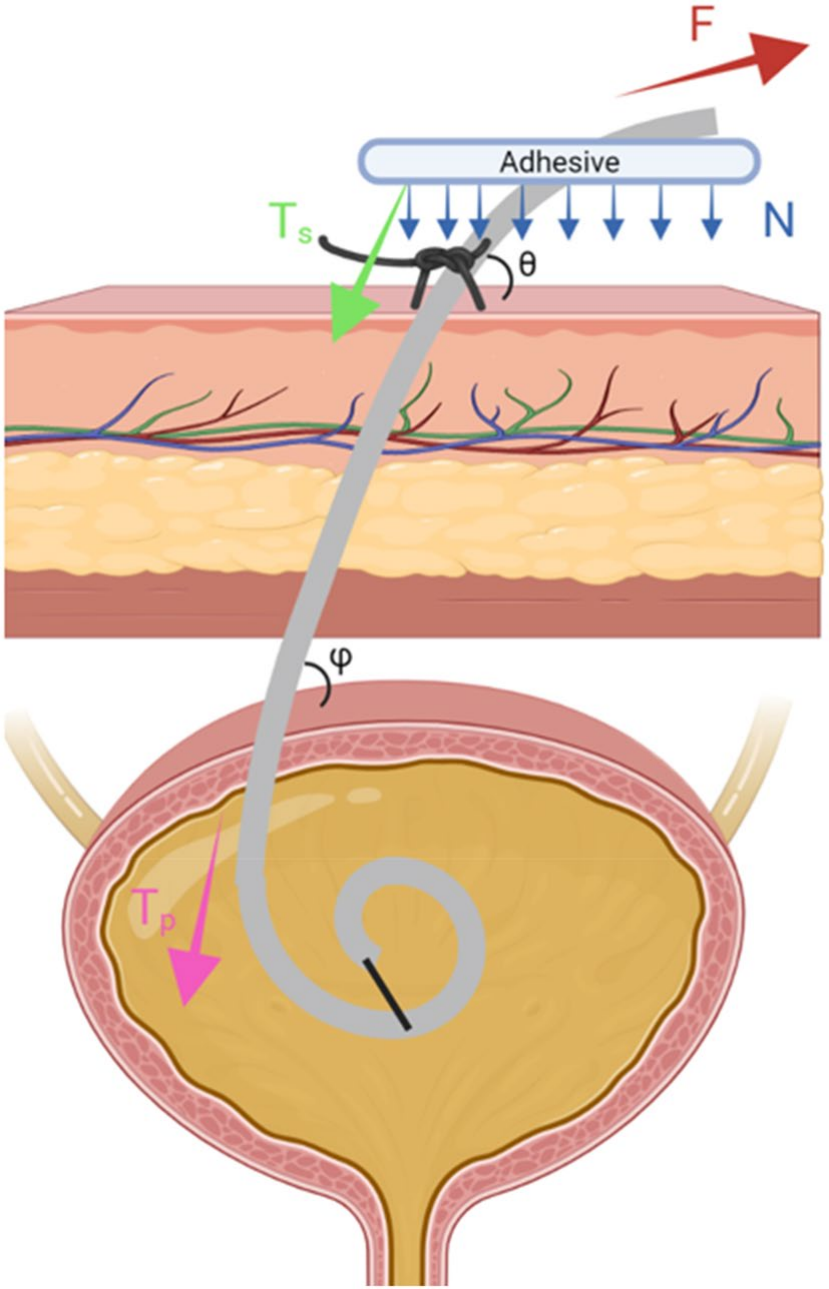
A free body diagram describing the forces that resist dislodgement of a drainage catheter. Force F represents the pulling force imparted on the drainage catheter externally. Resisting this, the adhesive forces (N), drain suture tension (T_s_), and the pigtail tension (T_p_) all act in opposition to the pulling force, and these are overcome when a dislodgement event occurs. Illustration produced using www.BioRender.com Individual License.

The current strategies for catheter fixation are inadequate^8^ and there is a paucity of data describing the forces encountered by a drainage catheter during normal use. A force-body diagram was utilized in order to understand the individual elements of a standardly secured drainage catheter (Fig. 2). The standard of care for drain securement relies in part on two primary elements: internal and external fixation components. Internal fixation components are intrinsic to the type of catheter placed, which include an end pigtail—with resistive force provided by the tensile strength of an inner securement string and by the elastic modulus of the tube—or from a retention balloon with resistance based on the size and pressure of the balloon. External fixation is operator dependent, with providers typically using a drain stitch or “sandal” stitch, which secures the catheter to the patient’s skin. Beyond standard of care, multiple groups have developed skin securement devices, such as the StatLock, which clamp around the drainage catheter and utilize an adhesive component to stick to the patient’s skin, adding an in-line linear resistive component. Further, some groups have developed a break-away device at the drainage catheter hub that allows the catheter to linearly decouple when a dislodging force is applied.

Current catheter securement modalities have several drawbacks. The most commonly used strategy of a drain suture induces tissue trauma and does not prevent the transmission of accidental pull forces to the internal portion of drain catheters. Other proprietary external catheter fixation mechanisms improve on patient comfort but sacrifice the security of both external and internal positioning, often by allowing some degree of sliding of the catheter tubing. Several groups have described the use of a linear breakaway device to reduce dislodgement rates^7^. However, the lack of a valve in currently available breakaway devices results in bodily fluid staining clothes or patients, and makes thoracic applications implausible given the risk of pneumothorax. The ideal drainage securement device may have elements that reduce the amount of extended tubing hanging from the patient, increase the friction safety factor when a catheter is pulled, and incorporate a valve-containing breakaway device that decouples following application of a multidirectional dislodgement force.

**Figure 3:**
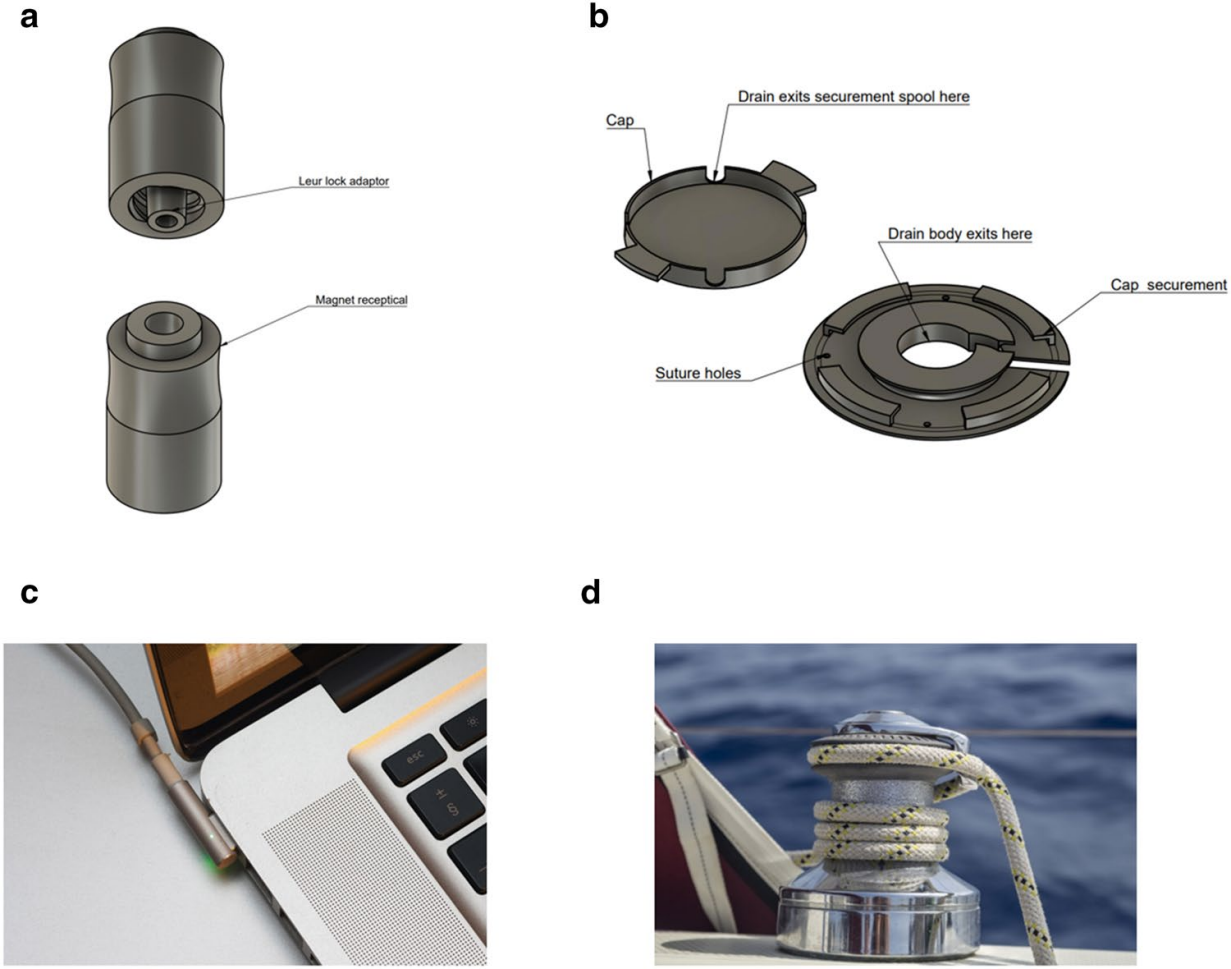
Inspiration of a proposed securement device from sailing and tech industries—pictured are the quick-release unit (QR, **a**) and securement component (SC, **b**). The QR is placed in-line with the catheter and does not allow internal transmission of forces great enough for dislodgement. A percutaneous catheter is wound around the SC, which is adhered to the body and prevents migration of the catheter. See supplemental video 1 which demonstrates an animated model of the device in action. The inspiration for the QR draws from current technology solutions for a safe power supply connection (**c**), and the inspiration for the SC is inspired from sailing winches, which also employ the Capstan principle (**d**). Figure 3a-b were produced using Fusion360 V.2.0.15299. Figure 3c was produced by Ashley Pomeroy (see acknowledgment)—Own work, CC BY-SA 4.0, Fig. 3d was produced by George Hodan (see acknowledgement)–—wn work, CC0.

In searching for solutions, the authors took inspiration from other industries. Increasing the friction of a line in a contained space has long been solved in the boating industry through the use of capstans where winding of a non-elastic line around a central column dramatically increases its resistive frictional force with a relatively small holding force (Fig. 3D). Quick release mechanisms are employed across daily life including gas station line safety break mechanisms or the MagSafe power cord (Apple Inc., Cupertino, CA), which disconnects from the laptop computer when the charging cord is pulled (Fig. 3C).

Utilizing these inspirations, in this study, ex vivo and in vivo models were used to determine the forces that characterize ACD. The authors analyzed the forces necessary to dislodge a drainage catheter and developed a novel, 3D-printed securement device specifically designed to limit the possibility of ACD.

## Materials and methods

### Device development and characterization

**Figure 4:**
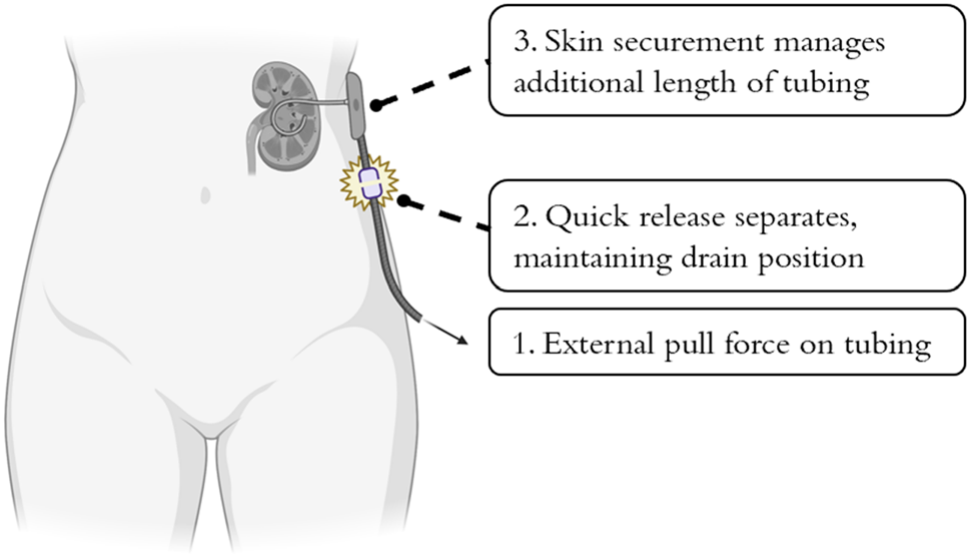
Conceptual demonstration of the novel, 3D-printed securement device—pictured are a kidney, percutaneous nephrostomy tube, and the securement device. The device noninvasively and safely prevents migration or dislodgement of the drain by reducing the profile of external drain tubing and eliminating the transmission of dislodgement-level pull forces. Figure was produced using www.BioRender.com Individual License and Microsoft PowerPoint V16.72.

Computer-aided design (CAD) software (Fusion 360, Autodesk Inc., San Rafael, CA), a Form 3 3D printer (Formlabs Inc., Somerville, MA), and a MakerBot Replicator 3D printer (MakerBot, Brooklyn, NY) were used in the iterative design and production of the device. We based development on a design thinking methodology and research infrastructure as previously described by our group^9–11^. The proposed device has three main components: (1) a skin-level catheter securement device which manages the external length of drainage catheter tubing and increases the frictional resistive force, (2) an adhesive component which affixes the skin level catheter securement device to the patient’s skin, and (3) a valved Luer lock compatible quick release mechanism that is interposed between the drainage catheter hub and drainage bag tubing (Fig. 3a-b, supplemental video 1), which detaches when a sufficient pull force is applied to the external catheter tubing and ensures the critical, internal drainage catheter component remains in place (Fig. 4).

Using 3D printing, the proposed skin-level catheter securement and quick-release device were generated. The components were fabricated using a combination of thermoplastic polyurethane (TPU), polylactic acid (PLA), and photopolymer resin. The flexible TPU base of the catheter securement component enabled flush skin-level apposition of the device and secured drainage catheters with a variable external length ranging from 4 to 30 cm near the site of percutaneous entry. The skin level catheter securement device has a surface area of 32.5 cm^2^. The drainage catheter is coiled around the central spool of the securement device and keeps the drainage catheter tubing within 6 mm of the patient’s skin. The skin-level catheter securement device was hand sutured to the adhesive component of the Hollister ostomy appliance, which was chosen for its adhesion, conformability, porosity, and fluid resistance. The quick-release component, composed of two halves, is designed to be connected to any Luer fitting at the terminal end of the drainage catheter, and the second half is designed to connect to the Luer fitting of any stopcock or drainage bag tubing. Neodymium ring magnets were utilized as the coupling force between the two halves of the quick release. A stop valve is incorporated into the quick release device to prevent fluid leakage when the quick release components are uncoupled during a dislodgement event.

### Force body diagram analysis

Using the force body diagram (Fig. 2), important contributing forces to ACD in the current standard of care were identified, including the force required to break a standard 2–0 polypropylene drain suture (Prolene, Ethicon, Inc., Raritan, NJ), the force required to break the pigtail-forming string within the drainage catheter, and the force required to remove the adhesive component of an ostomy appliance (Hollister Inc., Libertyville, IL), an appropriate surrogate for a non-woven skin adhesive. Appropriate force analysis equations were generated from this analysis. We then added the Capstan component to mathematically model the impact of our proposed skin-level securement device.

### Ex Vivo component force analysis of drain dislodgement

The component forces noted above which resist drainage catheter dislodgement were determined empirically. Additionally, the force required to remove a drainage catheter secured by a linear fixation device, the force required to decouple the proposed quick release component, and the resistive force of the proposed skin level catheter securement device were also tested. Forces were applied with a McMaster-Carr Tension and Compression Digital Force Gauge (McMaster-Carr, Princeton, NJ) by one operator until device failure and data was exported to Excel (Microsoft, Redmond, WA). At minimum, three trials from each component were obtained. By then employing our mathematical model with the necessary variables established empirically, the idealized peak force that a drainage catheter can resist before dislodgement with the presently reported skin-level securement device and the proposed skin-level securement was calculated. The idealized peak force was compared to the experimentally derived force required to fully remove a drainage catheter to validate whether the capstan equation applied to the proposed device in practice.

### In vivo assessment of drain dislodgement

The study used three female Yorkshire pigs, with weights between 40 and 50 kg, for testing of the device. Animals remained under general anesthesia for the duration of the experiment and were euthanized at the end of the experiment with inhaled Isoflurane 1–2% followed by 100 mg/kg Phenobarbital IV. All experimental protocols for this portion of the study were approved by the Institutional Animal Care and Use Committee (IACUC) at the Massachusetts General Hospital (MGH) under protocol #2021N000227, and all methods were completed in accordance with IACUC guidelines and regulations. Methods and results are reported in accordance with ARRIVE guidelines. 10.2 Fr Dawson Mueller (DM) drainage catheters (Cook Medical, Bloomington, IN) were aseptically inserted into the porcine bladder using standard trocar technique and ultrasound guidance. Drainage catheter pigtails were formed in the standard fashion. Drainage catheters were secured in three ways: without any securement, with a standard 2–0 polypropylene drain suture, and with the described skin-level securement adhered to the animal’s skin. These trials were conducted in sequence on the same pigs to reduce confounding. Initial catheter position was confirmed either fluoroscopically or with computed tomography (CT). A tensile force great enough to fully dislodge the catheters was applied with a digital force gauge and data were exported to Excel. Initial and post-pull catheter position was determined fluoroscopically.

## Results

### Force body diagram analysis

The forces on a drainage catheter can be decomposed into two directions, x and y, as a sum of the force on the drain itself (F_d_), tension on the suture (T_s_), tension on the pigtail (T_p_), and any linear adhesive normal force (Nl adhesive * u) in the x or y direction. (Fig. 2).

At stability (F = ma = 0),

In the x-direction, we find the system can be modeled as:

$$F = ma = 0 \rightarrow F_{d} *cos\left( \theta \right) = T_{s } cos\left( \theta \right) + T_{p} cos\left( \phi \right) + \left( {Nl_{adhesivex} *\mu_{adhesive} } \right)$$

In the y-direction, similarly, we find the system can be modeled as:

$$F = ma = 0 \rightarrow F_{d} *sin\left( \theta \right) = T_{s } sin\left( \theta \right) + T_{p} sin\left( \phi \right) + \left( {Nl_{adhesivey} *\mu_{adhesive} } \right)$$

The simplified model has several limiting assumptions. Forces on the drain followed a stepwise break function, i.e., the adhesive is in line with the suture/pig-tail drain, and that each of these subcomponents have a certain breakpoint at which they no longer contribute. Force on the drain at any point is dynamic, both externally and internally, resulting in each force being a function of time.

The addition of a capstan component in the skin level catheter securement device changes these formulas as follows with the frictional force between catheter and securement device now described with an exponential relationship $e^{\mu \alpha }$, previously $\left( {Nl_{adhesivey} *\mu_{adhesive} } \right)$.

At stability (F = ma = 0),

In the x-direction, we find the system can be modeled as:

$$F = ma = 0 \rightarrow F_{d} *cos\left( \theta \right) = e^{\mu \alpha } \left( {T_{s } cos\left( \theta \right) + T_{p} cos\left( \phi \right)} \right)$$

In the y-direction, similarly, we find the system can be modeled as:

$$F = ma = 0 \rightarrow F_{d} *sin\left( \theta \right) = e^{\mu \alpha } (T_{s } sin\left( \theta \right) + T_{p} sin\left( \phi \right))$$

Functionally, applying the capstan equation to the drain force body diagram analysis provides a multiplicative effect of the adhesive towards the sum force of the T_s_ (suture) and pigtail internal string (T_p_). This predicts a significantly higher resistive force for a coiled adhesive design over a standard linear one.

### Ex vivo component force analysis of drain dislodgement

**Figure 5:**
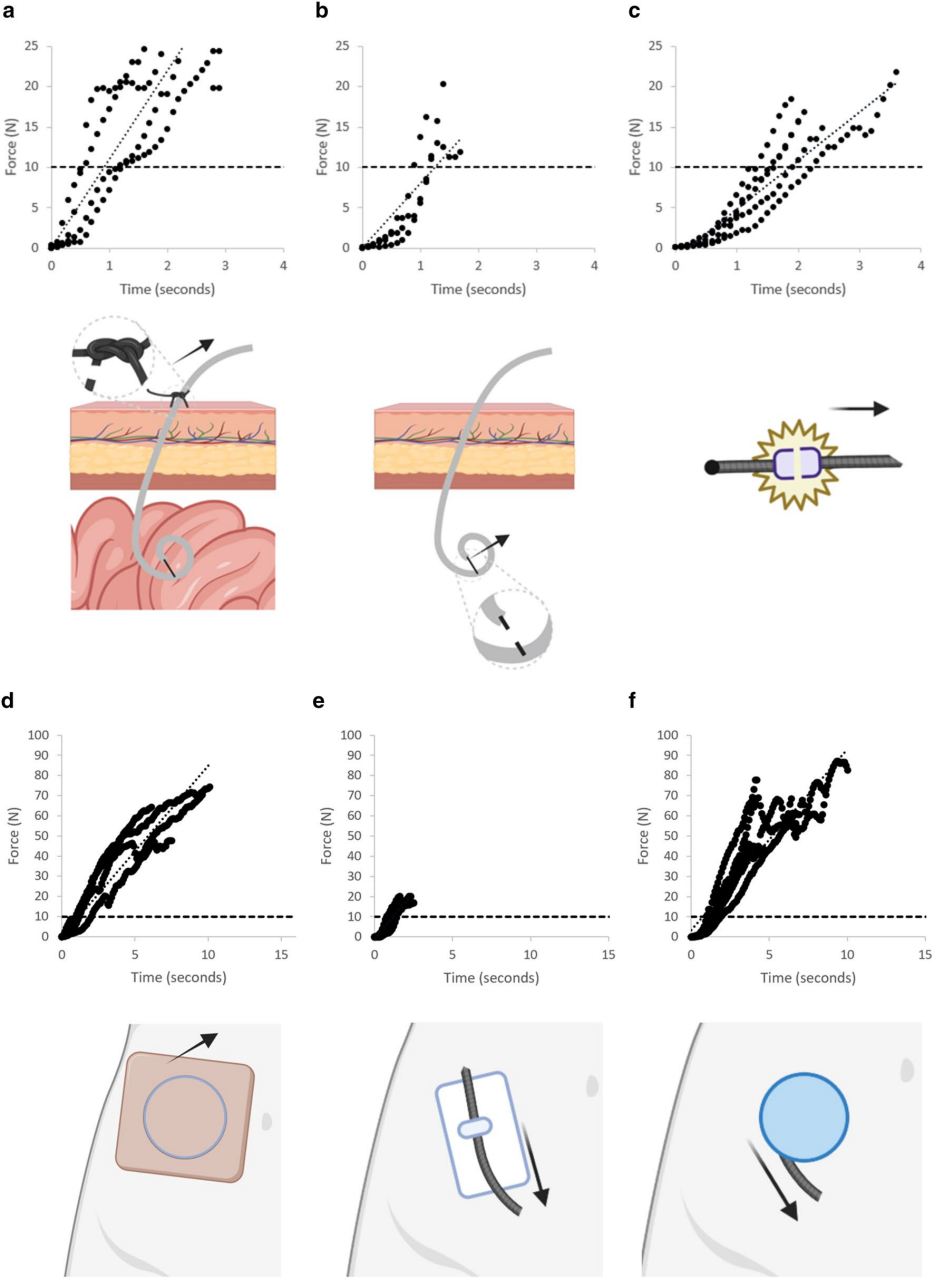
Ex vivo force measurements for dislodgements: the force to break a drain stitch (**a**), the force to break the inner securement of the drainage catheter pigtail (**b**), the force to disengage the quick-release component (**c**), the force to remove an ostomy adhesive (**d**), the force resisted by a linear fixation device (**e**), and the force resisted by the proposed device which implements a spooled design (**f**). The black dotted line refers to the average force for a 1 L amount of fluid at 1 g (acceleration ~ 9.8 m/s^2^), about two times the average volume held by drainage bags, and the clinical utility force required to be kept with accidental dislodgement. Illustrations produced using www.BioRender.com Individual License.

In order to apply the simulation, we empirically measured the above specific forces, T_s_, T_p_, and the linear adhesive normal force of an ostomy appliance. The 2–0 polypropylene drainage suture failed at 32.0 ± 5.9 N (n = 4) (Fig. 5a), the pigtail failed at 16.5 ± 3.6 N (n = 3) (Fig. 5b), and the ostomy appliance adhesive failed at 64.3 ± 11.7 N (n = 4) for parallel to skin pull forces (Fig. 5d). Assuming a linear system, the theoretical maximum force resisted by the standard of care is the sum of the drainage catheter suture and pigtail forces: 48.8 ± 7.1 N. We then analyzed the ex vivo predicted components of using a coiled instead of linear friction model. With one revolution around the spool, it was able to resist 78.5 ± 8.4 N (n = 5) forces (Fig. 5f). Conversely, a linear fixation system resisted 19.1 ± 1.8 N (n = 3) prior to the drain slipping out through the linear fixation system (the adhesive itself did not fail) (Fig. 5e). The quick release mechanism decoupled at 17.5 ± 2.7 N (n = 4) (Fig. 5c), providing an adequate safety margin to dislodgement. An average drainage bag can be expected to hold between 500 to 1000 mL, or roughly 5–10 N of expected force when hanging by gravity. An ideal quick release mechanism was thus determined to decouple 15–20 N allowing for a ~ 2 × safety factor to inappropriate decoupling during daily use, while also allowing for a 3–5 × safety factor compared to the force required to break either a catheter drain stitch or remove an adhesive device.

### In vivo assessment of drain dislodgement

**Figure 6:**
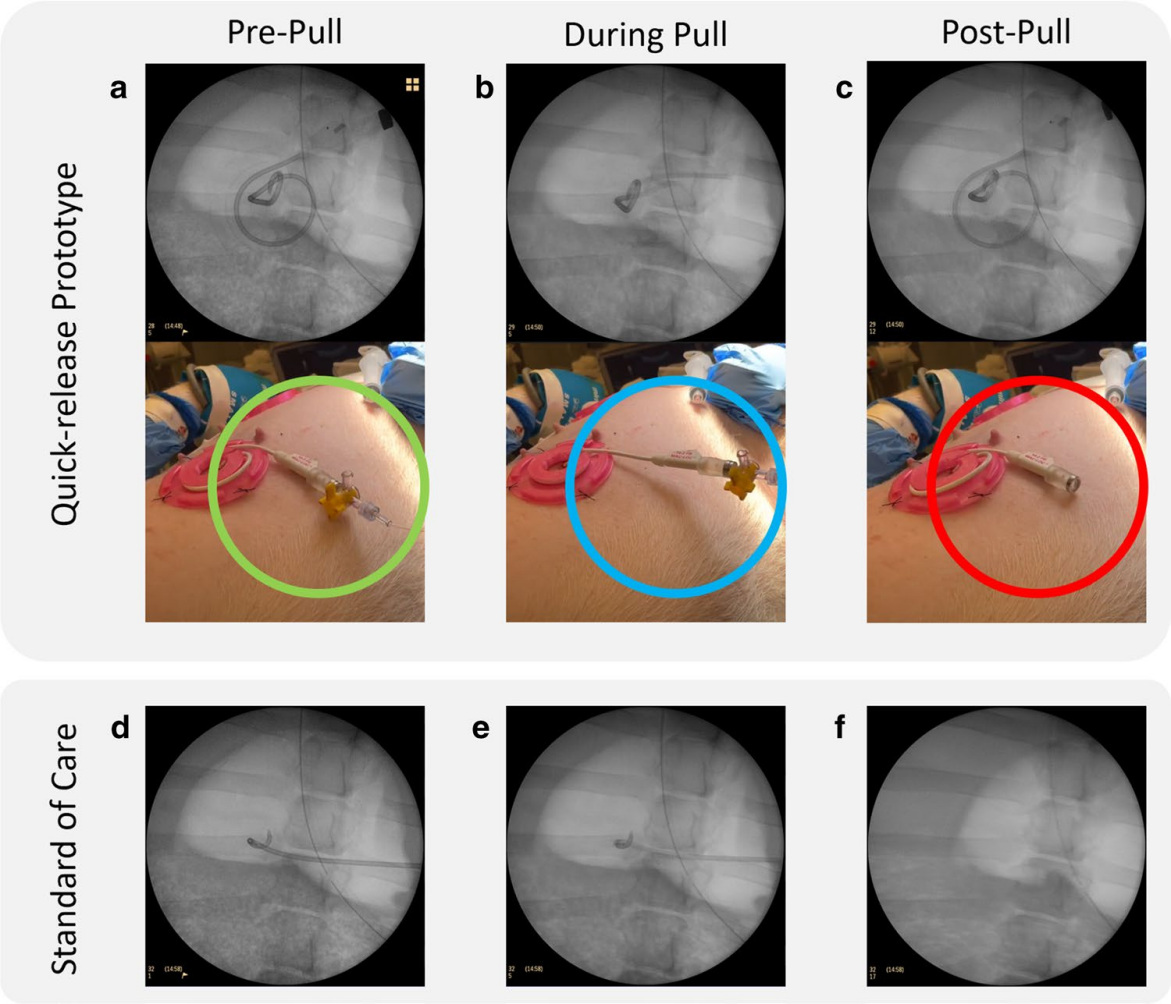
In vivo dislodgement and fluoroscopic evaluation—Following fluoroscopic drain placement, the impacts of pull forces on internal drain placement were compared between our novel securement component (SC), with a break away mechanism, and a drain stitch, the current standard of care. Fluoroscopy demonstrates proper drain positioning (**a**) with our novel SC attached and no pull force applied (green circle). A pull force is applied (blue circle) with maintenance of the drain pigtail position (**b**). The pull force was increased until the SC break away mechanism reached its detachment threshold (red circle) throughout which time the drain pigtail remains in an identical location (**c**). In contrast, a drain was placed under fluoroscopy and secured using a drain stitch (**d**). A pull force was applied with partial internal migration of the drain tip (**e**). The pull force was increased until failure of the drain stitch resulting in complete dislodgement of the drain (**f**). Figure was produced using Microsoft PowerPoint V16.72.

**Figure 7:**
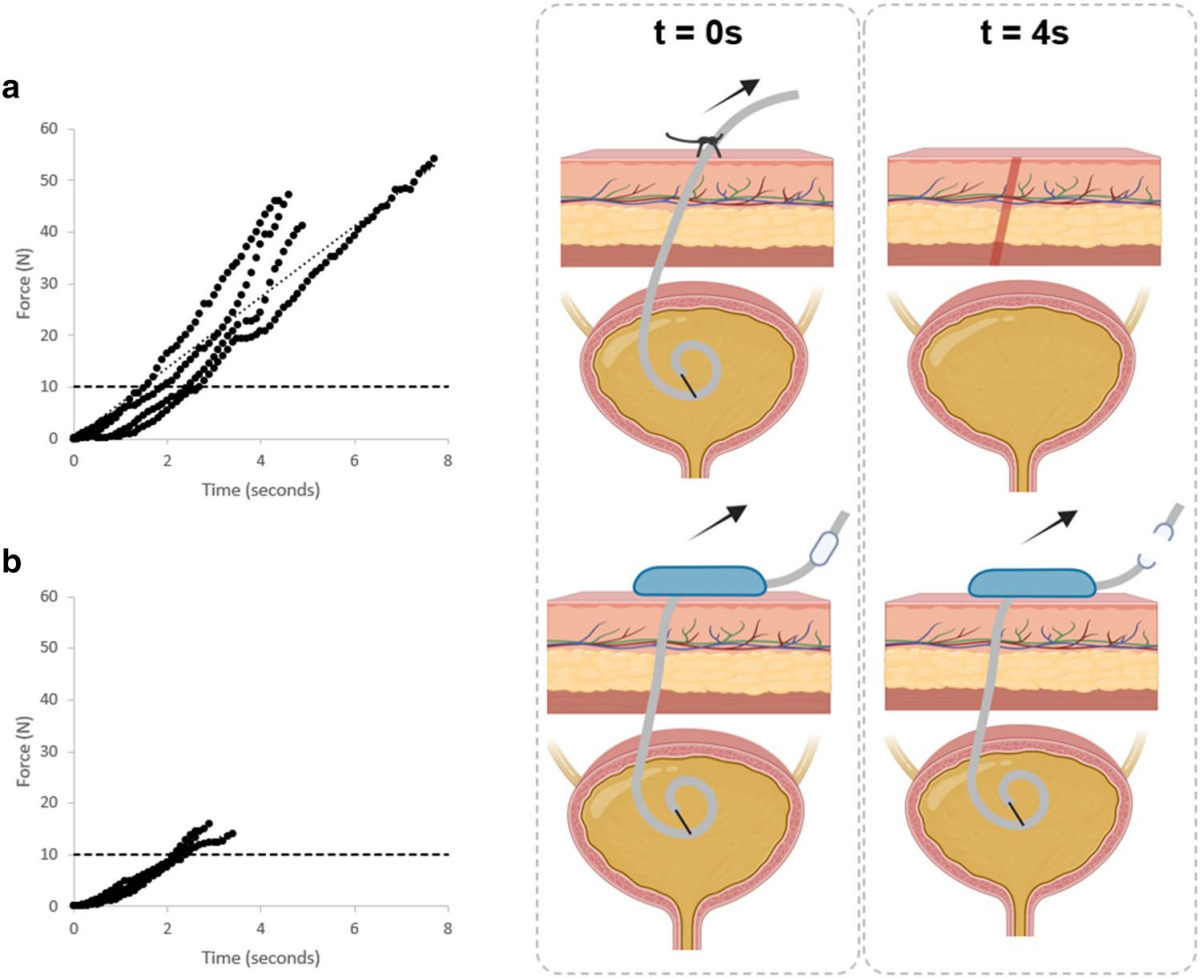
In vivo forces resisted by the standard of care and proposed device. Under the standard of care (**a**), forces peak at the drain stitch breaking (47.7 ± 4.6 N), resulting in complete dislodgement of the drain. Conversely, when implementing the proposed device (**b**), the drain first coils around the spool, removing slack. Then, once forces reach a sufficient level, the quick-release detached, keeping the drain in place. The maximum force resisted by the quick-release device was 15.1 ± 0.7 N. Illustrations produced using www.BioRender.com Individual License.

In the *in-vivo* model, without any catheter securement, the drainage catheter was displaced with unregistered forces (< 1 N). A catheter secured with a drain stitch and internal pigtail was dislodged with a peak force of 47.7 ± 4.6 N (n = 4). A drainage catheter fully secured by the proposed device is pictured in Fig. 6a–c (sans cap for illustrative purposes). When a pull force was applied, the catheter wound more tightly around the device’s central column, as slack was removed with tension. Eventually, the force became large enough for the quick-release unit to disconnect. The critical internal pigtail component of the drainage catheter remained in unchanged position throughout the pull event (Fig. 6a–c). In contrast, the pigtail was observed to have displaced under fluoroscopy when secured with only a drain suture (Fig. 6d–f). Overall, the proposed securement device detached at 15.1 ± 0.7 N (n = 3) and resulted in no migrated or dislodged catheters (Fig. 7). In the suprapubic model, urine continued to drain from the bladder while the catheter was coiled within the skin level securement device, which was engineered to prevent catheter kinking.

## Discussion

In this proof-of-concept study, we have taken steps to better understand the conditions under which drainage catheters dislodge. We used this data to inspire a new approach to limiting drain dislodgement, which was successful when tested in a swine model.

With over 3.2 million percutaneous drainage catheters placed in the US annually, ACD represents a significant source for patient morbidity and mortality as well as a burden on healthcare systems. Prior reports illustrate an ACD incidence of 4.5%–12.6% for abdominal drains^3,12,13^, 10.2%-12.8% for percutaneous gastrostomy catheters^2,7,14^, and 6.6%-9.2% for thoracic drains^6,15,16^, with a single prospective RCT subanalysis reporting rates of ACD for thoracic drains as high as 28%-42%^17^. Including the average cost of ambulance transportation, adult emergency room visits, and non-invasive imaging, the U.S. healthcare system conservatively estimated to spend over $2.5 billion on the management of dislodged percutaneous drainage catheters annually. This figure does not include the significant cost associated with repositioning and replacement procedures or potential hospital admission. Beyond the costs of replacement, significant morbidity is caused by ACD; dislodgement of percutaneous gastrostomy catheters, especially prior to formation of a mature gastrocutaneous fistula, may lead to peritonitis requiring surgical consultation^4,7^ and dislodgement of a percutaneous chest or abdominal drainage catheter may result in abscess/pneumothorax reaccumulation, hemodynamic compromise, or death^3^. This study establishes the forces that lead to dislodgement of drainage catheters secured with standard of care and proposes a novel catheter securement and quick-release system that better mitigates this ubiquitous complication.

While several external and internal fixation strategies exist, a method to sufficiently maintain drainage catheter position during a dislodgement event has not gained widespread use. A sufficient force will always overcome a catheter fixation device. This necessitates the use of a quick-release component to interrupt dislodgement forces from being transmitted to the critical internal component of a catheter. Applying lessons from the electronics and fuel industries to develop a valved approach builds on the lessons by other linear non-valved groups that have shown benefit to reduce dislodgement. The proposed valved quick release allows a solution for even externally short drainage catheters, where external length may be less than 4 cm.

Current fixation modalities leave a significant length (5–15 cm) of external drainage catheter unmanaged, thus increasing the risk of partial or complete dislodgement. This risk would be maintained even with the use of a quick-release device as pull forces proximal to the quick-release would still result in dislodgement. Given the enormity of ACD with these devices on the market, a new approach that secures the entire external drain close to the skin and allows for the interruption of dislodgement forces is imperative. Contrary to other fixation strategies, the herein described securement device delivers a low-profile solution to this problem. By employing inspiration from the sailing industry in applying and miniaturizing the capstan equation, we aim to mitigate external forces on the drain and pigtail while managing the entire external drain length. The empirical results of a spiral coil demonstrated a fourfold increase in drain safety compared to a linear adhesive model. This coiled design further has the advantage of maintaining drain integrity without kinking the drain by bending or compressing it. Numerous iterations of prototypes and calibration of the quick release mechanism enabled us to successfully achieve our goal of withstanding normal daily forces, defined as the 5-10N required to hold a 0.5-1L drainage bag of fluid, while ensuring consistent catheter position. Additionally, the design of both the skin level securement and quick release components have demonstrated a low learning curve throughout initial ex vivo tests with patients, nursing staff, and clinicians. Following one demonstration, patients, nursing staff, and clinicians are facile at implementing this device. User feedback has been central to the iterative development of the device.

In this study, there are a number of limitations. The differences in cutaneous and subcutaneous tissue between swine and humans provide different resistances to the dislodgement of catheters. Furthermore, none of the experimental conditions fully embody the breadth of catheter dislodgement situations. The experiments represent a comparative moment in time between solutions rather than the longitudinal conditions that would be experienced by patients. Future studies are needed to demonstrate longitudinal effectiveness and safety in patients. Additionally, the materials and production techniques used were selected based on cost-effectiveness and availability, and future iterations will employ more tailored adhesives, more flexible and conforming materials, and better tolerances.

Within these parameters, we have demonstrated that a three-component device providing skin-level securement adhered to a patient’s skin and a quick-release detachment mechanism is a compelling solution to ubiquitously dislodged catheters. Further, the holding force amplification and drain management achieved by the spool design suggest more devices should consider utilizing the capstan principle when securing medical interventions. While we are initially using drainage catheters, this strategy can be translated to many percutaneous devices, including vascular catheters, whose dislodgement may result in bleeding or interruptions in critical medications. Prevention of dislodged percutaneous catheters is of significant benefit to patients.

### Supplementary Information

Supplementary Video 1.

Supplementary Legends.

## Data Availability

The datasets generated during and/or analyzed during the current study are available from the corresponding author on reasonable request.
