# Supplementary material for: Preventing inadvertent drain removal using a novel catheter securement device
Source: Sci Rep. 2023 Sep 26;13:16130. doi: 10.1038/s41598-023-37850-2 (PMC10522644; doi:10.1038/s41598-023-37850-2)
Supplement: Supplementary file 2 — Supplementary Legends. [file 41598_2023_37850_MOESM2_ESM.pdf]

Supplementary Video 1: Animated video of proposed catheter securement device.
